# Children’s experiences with conscious sedation in dental care: a Norwegian cross-sectional study

**DOI:** 10.2340/aos.v84.44807

**Published:** 2025-10-22

**Authors:** Regina Skavhellen Aarvik, Edel Jannecke Svendsen, Maren Lillehaug Agdal

**Affiliations:** a Institute of Health and Society, Faculty of Medicine, University of Oslo, Oslo, Norway; b Oral Health Centre of Expertise in Western Norway, Bergen, Norway; c Department of Nursing and Health Promotion, Oslo Metropolian University, Oslo, Norway; d Department of Research, Sunnaas Rehabilitation Hospital, Nesoddtangen, Norway

**Keywords:** Conscious sedation, pediatric dentistry, dental anxiety

## Abstract

**Objective:**

This study explores how 9- and 17-year-olds in Norway experience conscious sedation during dental treatment, examining its impact on treatment ease, perceived ability to refuse, and memory retention.

**Material and methods:**

A cross-sectional electronic questionnaire was distributed to 13,013 children and adolescents (6,686 9-year-olds and 6,327 17-year-olds) in the Public Dental Service of Hordaland County, Norway, in 2019. Response rates were 65.6% for 9-year-olds and 52.2% for 17-year-olds. The survey included validated instruments for dental fear and nonvalidated items assessing subjective sedation experiences. Data were analyzed using descriptive statistics, chi-squared tests, and Mann–Whitney *U*-tests.

**Results:**

Of the respondents, 13.6% (*n* = 596) of the 9-year-olds and 9.5% (*n* = 313) of the 17-year-olds reported having undergone dental treatment with conscious sedation. Among them, 67.4% felt sedation made treatment easier, while 8.5% found it difficult to refuse treatment under sedation. A total of 51.2% remembered the treatment, and memory retention was associated with higher levels of dental fear (*p* < 0.001).

**Conclusion:**

While conscious sedation is perceived to facilitate dental treatment for many children, a large proportion retain memories of the treatment, particularly those with high dental fear. This highlights the importance of understanding children’s subjective experiences to improve sedation practices.

## Introduction

For many children, dental treatment is not just a routine procedure but a distressing experience that evokes anxiety, physical discomfort, and a strong urge to avoid it altogether. Estimates suggest that 9–15% of children experience dental anxiety at levels that hinder their ability to receive dental treatment [1]. This fear often leads to dental avoidance, delayed or inadequate treatment, and long-term negative impacts on oral health [2]. Managing children with dental fear and anxiety is, therefore, crucial for fostering positive dental experiences and supporting lifelong oral health.

Conscious sedation is a widely used approach to support anxious children during dental treatment. A recent systematic review highlighted that midazolam and nitrous oxide are among the most used agents worldwide [3]. By administering sedative medications, conscious sedation aims to induce relaxation while maintaining the patient’s ability to respond to verbal instructions [4, 5]. For many children, sedation reduces anxiety, enabling them to complete necessary treatment [4]. Additionally, sedation may promote a sense of mastery during dental visits, fostering positive associations and building trust with dental care. However, the evidence of the efficacy of conscious sedation as a means to habituate the child to dental care and to reduce dental anxiety is often of low or critical quality, emphasizing the need for robust clinical protocols and further high-quality research [3].

A recent review underscores inconsistencies in sedation outcomes, reinforcing the need to understand children’s subjective experiences [6]. Additionally, evidence suggests that repeated exposure to sedation may negatively impact children’s behavior in future treatments [7]. These findings highlight the complexity of sedation outcomes, particularly regarding memory retention. The extent to which sedatives induce amnesia in pediatric patients remains uncertain, as some studies suggest that while sedation can impair explicit memory, it may not entirely prevent children from recalling negative emotions during treatment [8]. Despite these concerns, the European Academy of Paediatric Dentistry (EAPD) [4] recognizes that sedation is a necessary tool in pediatric dentistry when nonpharmacological strategies, such as communication and distraction, are insufficient. The guideline emphasizes the importance of tailoring sedation practices to each child’s individual needs and emotional responses, ensuring a balance between minimizing distress and maintaining patient safety.

Given the potential of sedation to enhance children’s dental treatment experiences, it is crucial to gain deeper insights into their experiences with conscious sedation during dental treatment. This study addresses this need by exploring how 9- and 17-year-olds in Norway experience conscious sedation during dental treatment. Specifically, the study examines three key aspects of dental treatment under conscious sedation: (1) whether children perceive that conscious sedation eases dental treatment, (2) their sense of control (perceived ability to refuse treatment), and (3) their memory of the treatment. Additionally, the study aims to identify potential associations between sedation experiences and levels of dental fear and anxiety.

## Materials and methods

### Study design and population

This cross-sectional retrospective study used an electronic questionnaire distributed to all 9-year-olds (*n* = 6,686) and 17-year-olds (*n* = 6,327) in the Public Dental Service (PDS) of Hordaland County, Norway, between October and December 2019. The inclusion criteria encompassed all 9- and 17-year-olds residing in the county and registered in the PDS system. However, only those who provided active consent and completed the questionnaire were included in the study. Nine-year-olds were considered old enough to provide meaningful self-reports based on their personal experiences. Seventeen-year-olds were included to obtain insight into cumulative dental experiences before leaving the PDS. This study is part of a larger project, and a previous publication also describes the selection of age groups and study population [9]. As the study employed a population-based design, no formal sample size calculation was performed. The anonymous nature of the questionnaire prevented analyses of nonresponders.

Invitations were sent by SMS; 17-year-olds received the invitation directly, while parents of 9-year-olds received it on behalf of their children, as required by Norwegian healthcare regulations for children under 16 years [10]. The message emphasized the study’s focus on the child’s subjective experiences, and parents were instructed to assist their children if necessary.

Each participant received one invitation and three reminders at 2, 6, and 8 weeks. The SMS contained a link to the questionnaire, estimated to take 10 min to complete, which also included an informed consent form. The survey was written in Norwegian. To encourage participation, one iPad was raffled in each age group. Ethical approval for the study was obtained from the Norwegian Centre for Research Data (#783349/2019) and the County Dental Officer in Hordaland County Municipality (now Vestland). In addition, the Data Protection Officer in Hordaland County Municipality assessed the study to ensure compliance with data protection regulations. The study adhered to the STROBE (Strengthening the Reporting of Observational Studies in Epidemiology) guidelines for cross-sectional studies (Appendix A).

Hordaland County, which includes Norway’s second-largest city, Bergen, was the third most populous county in 2019 [11]. The region’s population structure reflects the national demographic, and the median household income is comparable to the national average [12], making Hordaland representative for epidemiological research in Norway. The Norwegian PDS provides individually tailored, free-of-charge follow-up care for children and adolescents up to the age of 18 years [13]. Most dentists in the PDS are general practitioners, with only 1% being specialists in pediatric dentistry [14]. In Norway, oral midazolam is the recommended agent for conscious sedation in pediatric dentistry [15].

## Data collection and instruments

The questionnaire contained both validated and nonvalidated items. The validated measures included the Children’s Fear Survey Schedule – Dental Subscale (CFSS-DS) and the Intra-Oral Injection Fear Scale (IOIF-s). The CFSS-DS is a 15-item scale (sum score range: 15–75, cut-off score > 38) widely used to assess dental fear and anxiety [16]. The IOIF-s is a 12-item scale (sum score range: 12–60, cut-off score > 38) validated for Norwegian children aged 10 to 16 years, used to assess intra-oral injection fear [17]. After close consideration, we used the IOIF-s for 9- and 17-year-olds, since the age difference was slight, and the items were developmentally appropriate. The questionnaire was piloted in both age groups (*n* = 26 in each), with no reported difficulties.

The nonvalidated items aimed to capture children’s subjective experiences with conscious sedation. The Norwegian questionnaire used the term ‘relaxing medicine’ as a child-friendly synonym for ‘sedative medicine/conscious sedation’. Respondents who reported having received relaxing medicine (with response options: ‘yes’, ‘no’ or ‘do not know’) were directed to additional follow-up questions assessing their experience. These follow-up items were rated on a 5-point Likert scale ranging from ‘not at all’ to ‘very high degree’. The questions related to the experience of sedation were phrased as follows:

- ‘Did the relaxing medicine make it easier for you to undergo dental treatment?’
- ‘Did the relaxing medicine make it harder for you to refuse dental treatment?’
- ‘Do you remember the dental treatment you received after taking the relaxing medicine?’

## Statistical analyses

Data analysis was conducted using IBM SPSS Statistics for Windows version 29.0. Descriptive statistics were generated using the ‘Frequencies’ function. Group comparisons were performed using Mann–Whitney *U*-tests for continuous variables and chi-squared tests for categorical variables. When the expected frequency in any cell was below five, Fisher’s exact test was applied. Statistical significance was set at *p* < 0.05. All statistical outliers were retained, as they represented plausible response values within the expected scale range.

All participants who responded having experience with conscious sedation during dental treatment were included in the statistical analyses. The dichotomized variables were coded as follows: for the item with response options ‘yes/no/do not know’, a code of 0 was assigned to ‘no/do not know’ and a code of 1 was assigned to ‘yes’. For items with five-point response options, a code of 0 was assigned to ‘not at all/low degree/neither high nor low’ and a code of 1 was assigned to ‘high degree/very high degree’. Responses on Likert-scale items were dichotomized to facilitate interpretation and analysis. This approach was chosen to allow for a clear comparison between positive and negative evaluations. The option ‘exclude cases pairwise’ was chosen in all analyses with missing data, indicating that the respective cases were excluded only if they had missing data required for the specific analysis.

## Results

A total of 13,013 children and adolescents were invited to participate in this study, comprising 6,686 9-year-olds and 6,327 17-year-olds. The response rates were 65.6% for the 9-year-olds and 52.2% for the 17-year-olds. The response rates for individual questions in the survey reported for both age groups combined, ranged from 43.5% to 59.9%. Among the respondents, 13.6% (*n* = 596) of the 9-year-olds and 9.5% (*n* = 313) of the 17-year-olds reported having undergone conscious sedation during dental treatment, and these respondents formed the basis for the analyses presented in this article. The distribution of sex by age group is presented in Table 1. Since the ‘They’ category only included one individual, this response was excluded from further analyses.

**Table 1:** The distribution of sex according to age.

Age (years)	Girls (%)	Boys (%)	They	Total
9	123 (39.4)	189 (60.6)	1	312
17	260 (43.6)	336 (56.4)	0	596

**Figure 1:**
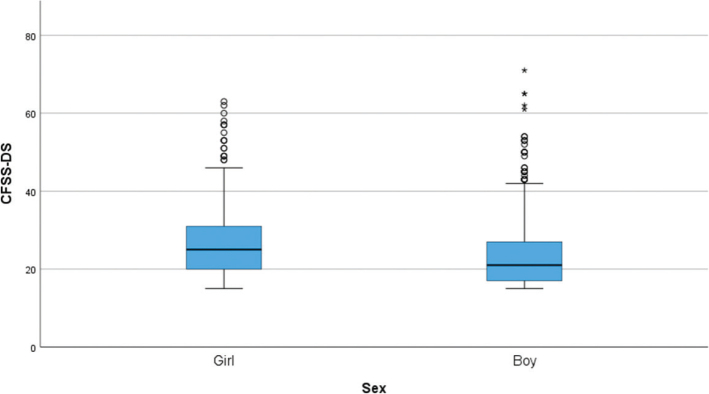
Boxplot illustrating dental fear scores (Children’s Fear Survey Schedule – Dental Subscale) for girls and boys. A Mann–Whitney U test showed that girls reported significantly higher scores than boys (p < 0.001).

**Figure 2:**
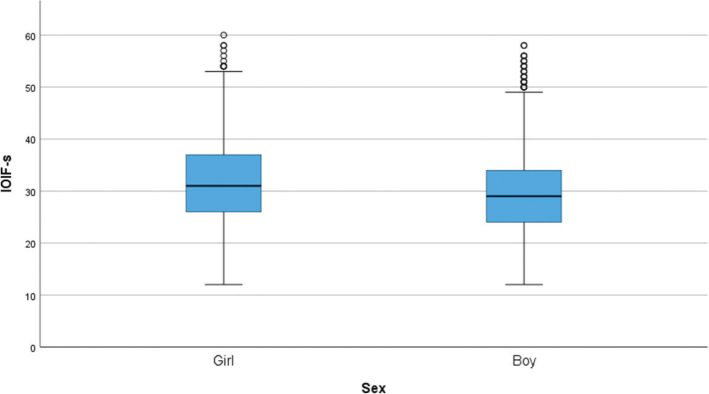
Boxplot showing intra-oral injection fear scores (IOIF-s) by sex. A Mann–Whitney U test showed that girls reported significantly higher intra-oral injection fear than boys (p < 0.001).

A total of 85 participants (9.4%) reported high dental fear, defined as a CFSS-DS score above 38. Table 2 shows that girls who have experienced dental treatment under conscious sedation generally score higher on dental fear and intra-oral injection fear, compared to boys. Figures 1 and 2 display boxplots of CFSS-DS and IOIF-s scores by sex, respectively. In both figures, girls show generally higher fear scores than boys.

**Table 2:** Mann–Whitney *U* tests evaluating the impact of sex on levels of the Children’s Fear Survey Schedule-Dental Subscale (CFSS-DS) and Intra-Oral Injection Fear-scale (IOIF-s) for participants who have experienced dental treatment under conscious sedation.

Study instrument	Girls	Boys	Total	*n*	Statistics
CFSS-DS	27.2 ± 9.1	23.8 ± 9.5	25.2 ± 9.5	908	*U* = 129855, *z* = 7.5, *p* < 0.001, *r* = 0.25
IOIF-s	32.0 ± 8.2	29.1 ± 7.9	30.5 ± 8.2	908	*U* = 117525.5, *z* = 6.5, *p* < 0.001, *r* = 0.22

**Figure 3:**
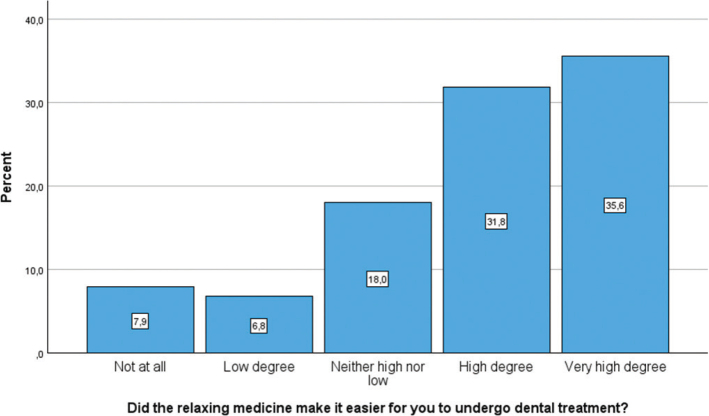
Bar chart showing participants’ responses to whether the relaxing medicine made dental treatment easier. Responses are shown across a five-point Likert scale.

**Figure 4:**
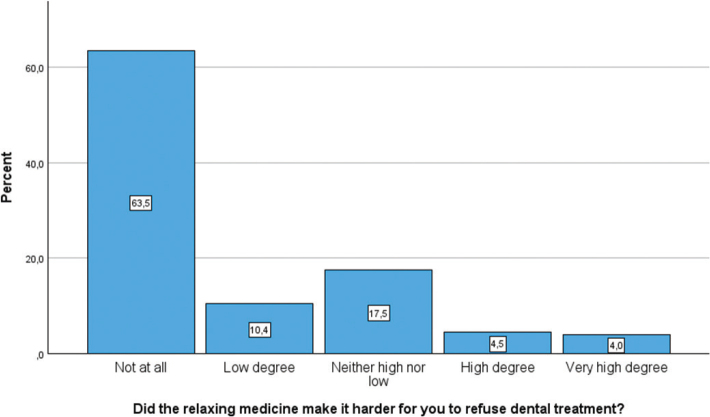
Bar chart showing participants’ responses to whether the relaxing medicine made it difficult to say no during dental treatment. Responses are shown across a five-point Likert scale.

**Figure 5:**
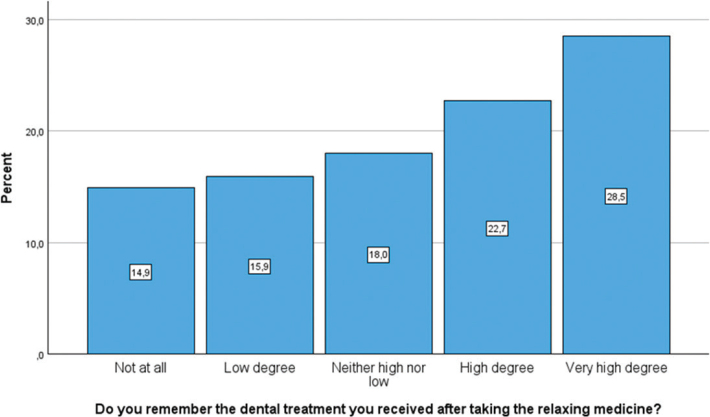
Bar chart presenting the degree to which participants remembered the dental treatment while sedated, rated on a five-point Likert scale.

Figures 3, 4, and 5 illustrate the distribution of participants’ experiences with different aspects of dental treatment after receiving relaxing medicine (conscious sedation). Measured by ‘high’ or a ‘very high degree’, 67.4% felt that conscious sedation had made it easier for them to receive dental treatment, while 8.5% experienced that conscious sedation made it harder to refuse dental treatment. In total, 51.2% remembered the sedated treatment in a ‘high’ or a ‘very high degree’.

A chi-square test showed a significant association, where participants with high dental fear (CFSS-DS > 38) were more likely to report that the relaxing medication made it easier to receive dental treatment compared to those with lower dental fear (*p* < 0.001). A chi-square test also indicated that participants with high dental fear found it significantly more difficult to refuse dental treatment when sedated, compared to participants with lower dental fear (*p* < 0.001).

**Figure 6:**
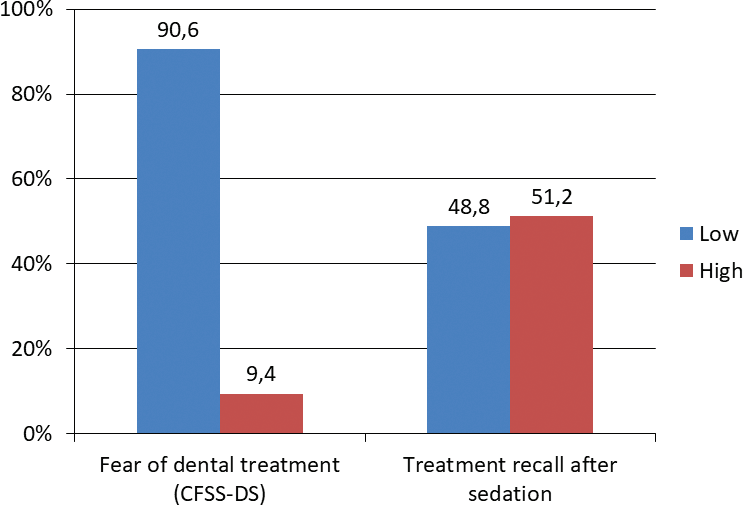
High and low dental anxiety (CFSS-DS) in 9- and 17-year-olds and high and low recall of dental treatment among the persons who had undergone treatment with sedation. Responses of ‘not at all’, ‘low degree’, and ‘neither high nor low’ were coded as ‘low’, while ‘high degree’ and ‘very high degree’ were coded as ‘high’.

The group who remembered dental treatment when being sedated had significantly higher dental fear compared to the group who did not remember dental treatment when being sedated (*p* = 0.001). Among the participants who reported one or several negative dental experiences (*n* = 97), 32.0% remembered dental treatment under conscious sedation in a ‘high’ or ‘very high degree’, while 14.9% did not remember the treatment. Figure 6 shows a bar chart presenting the percentage of children and adolescents’ responses to levels of dental fear (CFSS-DS) and their recall of treatment after conscious sedation.

## Discussion

This cross-sectional study provides important insights into pediatric patients’ experiences with conscious sedation during dental treatment. Most participants reported that conscious sedation made dental treatment easier, with a significant association between high levels of dental fear and the perception of easier treatment. However, these findings should be interpreted cautiously, as the study design does not permit conclusions about causality.

Our finding that 51.2% of children reported remembering the dental procedure despite conscious sedation is comparable to the memory retention rate reported in the systematic review and meta-analysis by Viana et al. [8], where approximately 47% of children who received benzodiazepines retained memory of the procedure (195 out of 418 participants). Their analysis of 54 randomized controlled trials showed that benzodiazepines (most commonly midazolam) significantly increase the likelihood of anterograde amnesia compared to placebo (RR = 3.10; 95% CI: 2.30–4.18). However, the review also highlighted that memory retention varies across individuals and settings. For instance, Jensen and Schröder found that 85% of sedated preschool children experienced amnesia, but those who retained memories were less likely to accept future dental care [18]. Conversely, qualitative research suggests that some dentists assume that children do not recall consciously sedated procedures and regard amnesia as a protective factor, even in potentially traumatic treatments [19]. The study relied on self-reported data, so the specific sedation agents could not be confirmed. Although oral midazolam is the most used sedative in the Norwegian PDS, the lack of clinical verification limits conclusions about agent-specific effects on memory and perceived control. Similarly, it is a limitation that the sedation provider could not be determined. In most cases, sedation is administered by general dentists within the Norwegian PDS, although some treatments may have involved pediatric dental specialists.

The clinical implications of this study’s findings, that many patients may remember dental treatment despite sedation and that approximately 15% did not feel the relaxing medicine made treatment easier, underscore the importance of effective behavioral guidance techniques. While conscious sedation is a useful tool for alleviating distress during dental treatment, it may not be sufficient for all children, particularly those with severe dental fear. Behavioral guidance techniques are considered a first-line approach for managing dental anxiety and should remain a priority before considering sedation [4]. In the present study, 13.6% of 9-year-olds and 9.5% of 17-year-olds reported having received conscious sedation. This represents a notable level of use within the PDS. Without national reference data on the use of sedation in pediatric dental care, it is not possible to conclude on the size of the figures in this study, and if the respondents differ from the general population. Conscious sedation is used both to reduce dental fear during treatment and to facilitate treatment in young patients and patients with behavioral or developmental challenges. Future studies should explore reasons and treatment strategies when sedation is used in pediatric dentistry.

Our finding that 67.4% perceived conscious sedation as helpful for enduring dental treatment supports previous research documenting its general effectiveness in pediatric populations [4, 5]. However, the child’s acceptance of the sedation is known to vary, and an important ethical consideration is the reduced ability of some children (8.5%) to express refusal during sedation. Such findings raise concerns about patient autonomy and highlight the need for clear explanations about what sedation can and cannot provide for the child, as inadequate communication might risk creating feelings of distrust in future dental treatments [20]. Ensuring that children retain the ability to signal discomfort and withdraw cooperation during sedation is critical. Clinicians should recognize that, even under sedation, discomfort or non-compliance may signal that the child is unwilling or unable to proceed with treatment. These findings underline the importance of adequate communication when providing conscious sedation to children, particularly in vulnerable or stressful clinical situations. This aligns with the findings by Lourenço-Matharu et al. [21], who showed that the most fearful children often remain behaviorally distressed and uncooperative, even when sedated with oral midazolam. Recent systematic reviews have emphasized the importance of empowering children during dental procedures by demonstrating the efficacy of psychological and behavioral techniques in reducing dental fear [22, 23]. In line with current EAPD guidelines [4], our findings support the use of conscious sedation as one element in a broader strategy. For highly anxious children, it may be particularly important to integrate behavior guidance techniques that promote a sense of control and emotional care before, during, and after sedation to ensure that the child can have positive feelings related to future dental treatments. Part of providing evidence-based care is also ensuring that the child’s emotional needs are met.

The strengths of this study include the large sample size and its broad representation of children and adolescents with experiences of conscious sedation. Conducting the study in Hordaland County, which reflects the national demographic and socioeconomic distribution, enhances the generalizability of the findings. However, the higher proportion of boys in the sample compared to the total survey population could introduce bias. It is also possible that boys are more likely to receive conscious sedation than girls although no prevalence studies have confirmed this in the current population. At the same time, girls in the present study reported higher levels of dental fear than boys, which may have influenced how they experienced and recalled treatment. While the response rates of 52.2% for the 17-year-olds and 65.6% for the 9-year-olds are acceptable, it remains important to interpret the findings with caution, as the experiences of nonresponders may differ from those who participated.

Although the questions provided valuable insights, the use of nonvalidated items to assess subjective experiences is a notable limitation. To enhance content validity, these questions were developed based on a comprehensive literature review conducted in collaboration with a medical librarian and informed by discussions with experts in pediatric dentistry and psychology. The research team included a pediatric dentist and a pediatric nurse specialist, both with doctoral degrees and extensive clinical and academic expertise in sedation and pediatric care. A test–retest reliability could not be assessed due to project constraints; therefore, the questions were pilot tested on both age groups to confirm that they were comprehensible and age appropriate, strengthening their face validity. Although the questions were pilot tested and carefully formulated for age appropriateness, the lack of psychometric validation may affect the reliability of the findings. Moreover, the use of Likert-scale items with potentially leading formulations, for instance, ‘Did the relaxing medicine make it easier to undergo dental treatment?’, could introduce response biases such as social desirability bias, particularly among younger respondents [24]. We also acknowledge that negatively framed questions, such as whether the medicine made treatment harder, may be similarly affected. More neutral formulations, for example ‘How did the medicine affect your experience of treatment?’ may be preferable in future studies. Further, in some analyses, the dichotomization of Likert-scale responses may have led to a loss of information, which should be considered when interpreting the results. However, this approach was chosen to simplify reporting and ensure that findings would be accessible to clinicians and policymakers. While open-ended questions might have provided more nuanced insight, the use of closed-ended items was necessary to ensure feasibility in a large-scale population-based survey. For a more detailed description of the methods and validation employed in the survey, refer to Aarvik et al. [9] and Aarvik [25]. Future studies should prioritize developing validated instruments for capturing children’s subjective dental and sedation experiences.

Another limitation is the potential influence of proxy reports. Although the survey targeted the children themselves, some responses may have involved input from their parents, particularly for younger participants. The retrospective design of the study also introduces the possibility of recall bias. There is a risk of social desirability bias, where participants may provide answers they think are expected rather than their genuine experiences. Nonetheless, previous research has demonstrated high concordance between self-reported data and medical records [26]. Depending on the type of information reported, self-reported data can be a reliable source of information in many contexts [26]. Notably, the cross-sectional design prevents conclusions about whether conscious sedation is used for the fearful child or if the child becomes afraid because of experience with sedation. However, repeated positive sedation experiences might reduce fear over time, as suggested in adult populations [27]. In summary, while the retrospective design and self-reported data present limitations, this study provides valuable insights into children’s experiences with conscious sedation and contributes to a foundation for further research in this field.

In conclusion, children and adolescents’ perspectives on dental treatment provide valuable information for improving clinical care. While conscious sedation was reported to ease treatment for most participants, more than half also retained memories of the procedure. The findings suggest that to support anxious pediatric patients, sedation should be complemented by behavior guidance techniques. Clear communication and collaboration between children, their parents, and dental professionals can foster trust and enhance the quality of care in pediatric dentistry. Further research is recommended, particularly longitudinal studies and the development of validated tools to assess children’s subjective experiences, to strengthen their voice and participation in pediatric dentistry.
